# Interplay Between Cortical and Neurocardiac Interoceptive Processes and its Association with Self-Reported Interoceptive Sensibility

**DOI:** 10.1007/s10548-025-01122-1

**Published:** 2025-06-03

**Authors:** Mariana Oliveira, Márcia da-Silva, Lídia Carvalho, A. Ribeiro-Carreira, Ana Rita Pereira, Adriana Sampaio, Joana Coutinho, Alberto J. González-Villar

**Affiliations:** Psychological Neuroscience Laboratory (PNL), Research Center in Psychology (CIPsi), School of Psychology, University of Minho, Braga, Portugal

**Keywords:** Interoception, Heart-Brain Axis, Heartbeat Evoked Potentials, Microstates

## Abstract

Interoception, the process of sensing and interpreting internal bodily signals, plays a crucial role in emotional regulation, decision-making, and overall well-being. This study aimed to investigate the relationship between self-reported interoceptive processes, assessed through the Body Perception Questionnaire (BPQ), and psychophysiological measures of interoception, including cardiac autonomic markers (HF-HRV and RMSSD), cortical processing of cardiac signals (heartbeat-evoked potentials, HEPs), and EEG microstates. We recorded EEG and ECG from 64 healthy volunteers during open-eyed resting state. A positive association was found between the Subdiaphragmatic Reactivity subscale of the BPQ and the coverage of microstate A, a spatial configuration linked to the activation of temporal brain regions, arousal, and sensory processing. No associations were observed between BPQ scores and cardiac measures or HEP amplitudes, suggesting that subjective reports may not align with psychophysiological indices of interoception. Associations were found between HEP amplitudes and microstates A and B, as well as between HRV measures and microstate D, highlighting potential links between autonomic functioning and brain activity during resting state. Although the BPQ is a widely used tool to assess interoceptive sensibility, it may not fully capture the complexity of this construct. These findings provide insight into the complex interplay between self-reported interoception and psychophysiological markers, while emphasizing the need for further research to clarify these relationships and their implications for emotional and cognitive processing.

## Introduction

The organism’s survival depends on its ability to adapt to an ever-changing environment. A key aspect of this adaptation is the optimization of energy regulation for both the brain and body (Bullmore and Sporns 2012), which relies on allostasis — the body’s ability to react to environmental changes, anticipate demands, and conceive biological plans to prepare for future needs. This anticipatory capacity is supported by the prediction and embedding of internal bodily states known as interoception (Schulkin and Sterling 2019).

Interoception is responsible for sensing, interpreting, and integrating the body’s physiological conditions (e.g., hunger, thirst, pain), thus providing a moment-to-moment map of the body’s internal *milieu* (Berntson and Khalsa 2021; Craig 2003). This capacity requires complex interactions between ascendent (afferent) and descendent (efferent) pathways that balance the body’s internal environment. It allows the prediction and control of sensory signals from peripheral systems such as cardiovascular, gastrointestinal, and respiratory functions (Levine 2007). At the functional level, interoception plays a role in decision-making, memory, social interaction, and emotional regulation (Adolfi et al. 2017). This complex construct was further divided into domains, including accuracy, which is related to objective precision in detecting internal bodily sensations; awareness, defined by our metacognitive capacity to be aware of our interoceptive accuracy; and sensibility, encompassing the self-perceived disposition to be internally focused on bodily sensations, which can be assessed, among other ways, through self-report measures (Garfinkel et al. 2015).

Given the critical role interoception plays in integrating physiological and emotional stimuli, recent literature supports that interoception is necessary for allostasis’s success (Barrett 2017; Pezzulo et al. 2015; Seth 2014). This led to the proposal of an integrated network called the Allostatic Interoceptive Network. In this approach, the top-down (autonomic) and the bottom-up (interoceptive) pathways are conceptualized as interacting bidirectionally, forming a dynamic exchange within the heart-brain axis (Ibanez & Northoff, 2023). This network can be characterized across multiple levels, encompassing multiple functional and neurophysiological mechanisms. For instance, a key physiological marker of this bidirectional interaction is Heart Rate Variability (HRV), which reflects the dynamic interplay of cortical interoceptive regions on autonomic regulation and, conversely, the impact of autonomic signals on higher-order interoceptive processes (Garret et al., 2023).

HRV seems to capture the flexibility and efficiency of the Autonomic Nervous System through the modulation of the vagus nerve, which underpins a better perception of internal bodily states (Thayer and Sternberg 2006). Evidence suggests an association between HRV and various dimensions of interoception, namely a positive correlation with interoceptive accuracy and sensibility (Lischke et al. 2021; Owens et al. 2018). While HRV seems to represent an index of the efficiency of allostatic interoceptive processes that allow the modulation of cardiac activity and, consequently, of different biological activities, heartbeat-evoked potentials (HEPs), despite ongoing debate, have been suggested by several studies to capture essential Allostatic Interoceptive Network brain hubs (Santamaría-García et al. 2024). The HEP is a scalp-recorded event-related potential that is time-locked to individuals’ heartbeats, aligned with the R-wave observed in the ECG, and reflects the cortical processing of cardiac activity (Coll et al. 2021). This neurophysiological marker has been connected with interoception and allostatic processes at both neurocardiac and autonomic levels and seems to allow the evaluation of the brain-heart axis communication (Virjee et al. 2024).

In addition to HRV and HEPs, EEG microstates offer another valuable window into the neural dynamics of interoception, serving as a useful tool for studying brain-heart interactions. Microstates show consistency across studies, with a standard set of four microstates, labeled “A-D” being identified in the majority of reports (Catrambone & Valenza, 2023; Michel and Koenig 2018; Tarailis et al. 2024). Although the cognitive processes underlying microstates remain debated, each microstate has been linked to specific functions: microstate A to auditory processing and arousal; B to visual processing, self-awareness, and autobiographical memory; C to self-reflection and personally significant information; and D to executive functions. An additional microstate E has been associated with interoceptive and emotional processing, linked to the salience network (Damborská et al. 2019; Khanna et al. 2015; Tarailis et al. 2024). These brief, stable patterns of brain activity are thought to reflect the brain’s transient configurations that facilitate the integration of sensory information, including internal bodily signals. As such, microstates provide a real-time snapshot of cortical processing associated with the brain’s response to physiological states (Lehmann et al. 2010). Their association with specific cognitive and affective processes (e.g., self-reflection, salience processing) makes them especially relevant to exploring interoception and the Allostatic Interoceptive Network.

Despite growing interest in the interplay between interoception, autonomic function, and brain dynamics, studies integrating self-reported interoceptive sensibility with both peripheral (e.g., HRV) and central (e.g., HEPs, EEG microstates) neurophysiological markers remain scarce, leaving a gap in our overall understanding of interoceptive processing, particularly within the framework of the Allostatic Interoceptive Network. Moreover, while some EEG microstates have recently been linked to interoceptive and affective processing, their role within the heart-brain axis remains underexplored. By simultaneously assessing HRV, HEPs, and microstate dynamics in relation to interoceptive sensibility in a healthy population, the present study aims to address this gap. This integrative approach provides a novel framework to investigate the multidimensional interplay between autonomic and cortical processes underlying interoceptive functioning, as well as its relationship with self-reported interoceptive sensibility. Specifically, we hypothesize that (1) self-reported interoceptive sensibility - measured via the Body Perception Questionnaire (BPQ) - will be associated with HRV, HEPs and microstates dynamics. Additionally, we anticipate that (2) HRV will be associated with microstates dynamics, reflecting the integration of autonomic and cortical processes. Finally, we hypothesize that (3) microstates parameters will be significantly associated with HEP amplitudes, providing further insight into the interplay between cortical and neurocardiac interoceptive processes.

## Methods and Materials

### Participants

Sixty-four healthy volunteers (33 male and 31 female), with ages between 18 and 40 years (*M* = 25.2; *SD* = 5.7) participated in this study. Participants were recruited through informal advertising and from the Credits Platform of the University. Exclusion criteria for the current sample were as follows: (1) age under 18 years; (2) the presence of any psychiatric, cardiac or neurological disorder; and (3) dependency or abuse of alcohol and/or drugs. Participants were informed about the experimental protocol and gave written informed consent before participating. The procedures complied with the principles expressed in the Declaration of Helsinki and received approval from the Ethics Committee for Social and Human Sciences of the University of Minho (CEICSH 030/2022).

### Psychological Measures

The initial assessment questionnaire included socio-demographic information (e.g., age, sex, nationality, education level) and questions related to the eligibility for this study.

After the EEG session, participants filled out the Portuguese version of the Body Perception Questionnaire (BPQ). The BPQ (Porges, 1993; Campos et al. 2021) aims to assess two main domains: body awareness and autonomic reactivity. The body awareness section is designed to assess individuals’ sensitivity to internal bodily functions (i.e., the interoceptive sensibility trait). On the other hand, the autonomic reactivity section consists of two subscales: the supradiaphragmatic symptoms subscale, which assesses sensations experienced above the diaphragm (e.g., heart rate, breathing, and upper body tension), and the subdiaphragmatic symptoms subscale, which evaluates symptoms experienced below the diaphragm (e.g., gastrointestinal sensations). Participants were asked to report how often they notice and become aware of certain physiological sensations described in each item, using a 5-point Likert scale (where 1 corresponds to “Never” and 5 to “Always”). Scores on the body awareness scale can range from 26 to 118, with higher scores indicating greater interoceptive sensibility. Scores on the autonomic reactivity scale range from 20 to 100, with higher scores indicating greater sensitivity and heightened awareness of autonomic physiological processes.

The Portuguese version of the Body Perception Questionnaire (BPQ) exhibits excellent internal consistency, with a Cronbach’s alpha coefficient of α = 0.96 for the Body Awareness subscale and α = 0.90 for the Autonomic Reactivity subscale.

### Procedure and Electrophysiological Recordings

Participants were accompanied to a properly isolated room, with little light and soundproof, where they were asked to sit in an armchair as comfortably as possible. All participants underwent a 4-minute resting-state period acquisition, during which they were instructed to maintain their gaze on a fixation cross presented centrally on a computer screen. They were asked to remain still, minimize blinking, and focus solely on the cross throughout the entire task.

Brain activity was recorded through a nylon cap with 64 active Ag/AgCl scalp electrodes (Biosemi ActiveTwo System, Amsterdam, The Netherlands). The electrode placements followed the guidelines of the international 10–10 system. The vertical (VEOG) and horizontal (HEOG) electrooculogram were recorded using an electrode placed below the left eye and 2 electrodes attached to the outer canthus of the eyes, respectively. Additionally, an electrode was placed on the tip of the nose for re-referencing. The electrocardiographic (ECG) activity was measured by positioning an electrode below the left clavicle. All recordings were referenced to the CMS (Common Mode Sense) and DRL (Driven Right Leg) Biosemi electrodes. Electrode impedances were kept between − 30 µV and 30 µV. The electrophysiological signals were recorded with a sampling rate of 512 Hz and an online bandpass filter ranging from 0.01 Hz to 100 Hz was applied.

### EEG Analysis

**Fig. 1:**
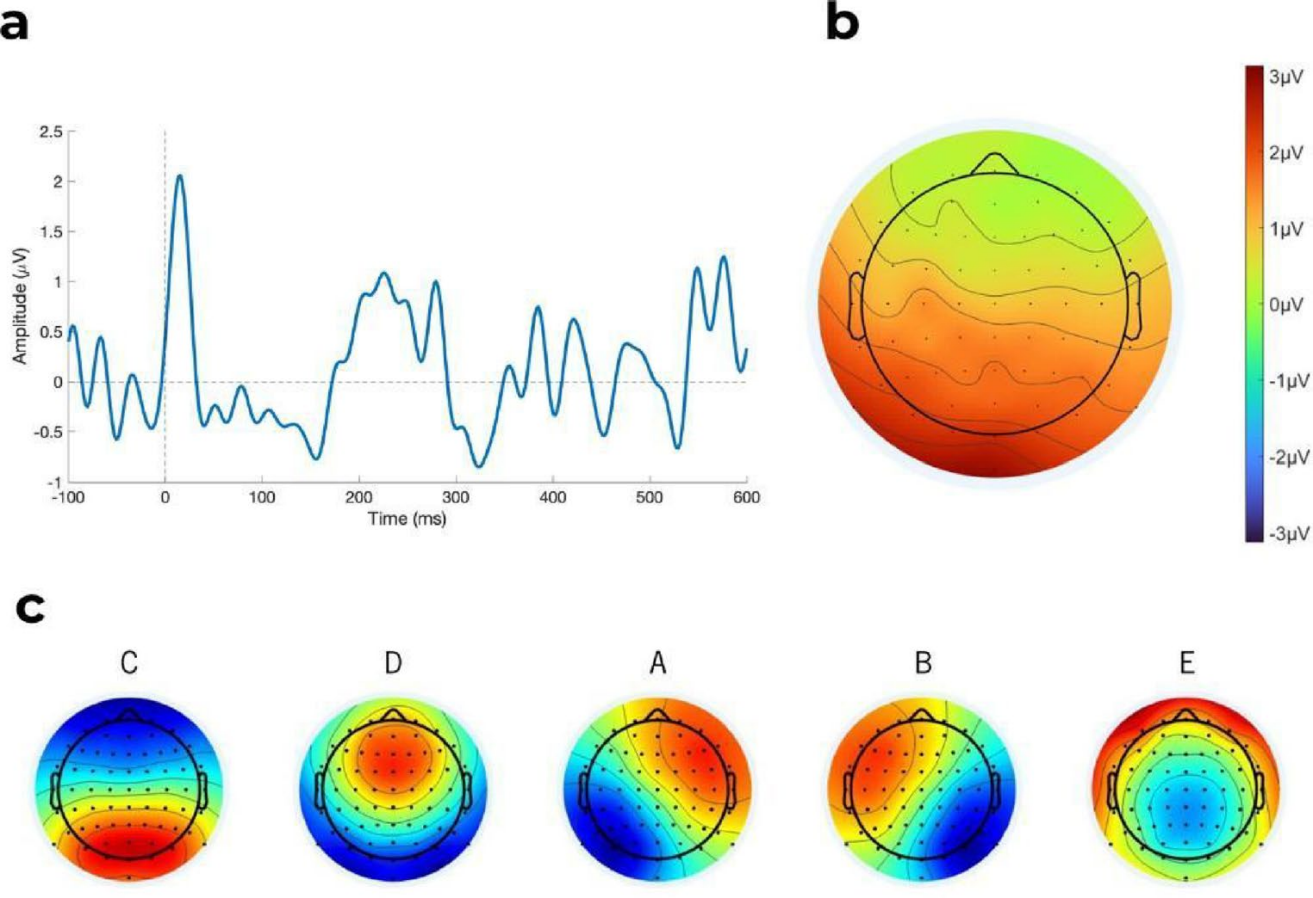
**a**: Time course of heartbeat-evoked potential at the selected region of interest. **b**: Topographical map of the heartbeat evoked potential amplitude within the 200–400 ms time window. **c**: Topographies of the 5 prototypes of microstates extracted

Offline EEG preprocessing was performed in Matlab (R2024b) with the EEGLAB toolbox (Delorme and Makeig 2004). After re-referencing to the tip of the nose, data were downsampled to 256 Hz and offline filtered between 0.1 and 30 Hz. EEG signals of malfunctioning electrodes were interpolated using the *Spherical Splines* method. For the analysis of Heartbeat Evoked Potentials (HEPs), epochs regarding the detected R-peak onset were extracted, with a time window of– 100 ms to 1000 ms relative to the onset of the stimulus. Trials were rejected if several channels showed non-stereotypical artifacts on visual inspection. Independent component analysis (ICA) was performed, enabling the identification and removal of components related to eye blinks, cardiac-field artifacts, saccades, or electrical noise, using the SASICA toolbox (Chaumon et al. 2015). After performing a baseline correction using the pre-stimulus interval (−90 to −10 ms regarding R-peak onset), average brain waves were computed in the R-triggered EEG segments. For this study, the maximum peak of the HEPs was extracted from the time window of 100 to 500 ms post-stimulus in the electrodes of a fronto-central region of interest (ROI): FC1, FC2, FC3, FC4, FC5, FC6, Cz, C1, C2, C3, and C4 (See Fig. 1a and b). This time window and ROI were selected based on previous literature (Coll et al. 2021).

### Microstate Analysis

To perform the microstate analyses we used the Microstate toolbox (Poulsen et al. 2018). EEG signals were segmented based on Global Field Power (GFP) and classified into distinct classes according to their topographical patterns. The datasets were normalized, and GFP peak maps were extracted from 1000 peaks per subject, ensuring a minimum peak distance of 10 ms. The optimal number of cluster maps was determined using the cross-validation criterion (Pascual-Marqui et al. 1995), comparing different classifications in a range from 3 to 7 clusters. The clustering method for classifying the microstates was the modified K-means algorithm (Pascual-Marqui et al. 1995). The convergence threshold was set to 10^−6^ and the maximum number of iterations was set to 1000. To address the stochastic nature of the algorithm, the classification process was repeated 50 times, and the solution with the lowest cross-validation criterion was selected.

After identifying the optimal number of microstate prototypes, these were back-fitted to all EEG recordings, disregarding polarity, as recommended for spontaneous EEG analysis (Poulsen et al. 2018). The back-fitting process employed the Global Map Dissimilarity index (Murray et al. 2008). Additionally, short segments of unstable topographies (less than 30 ms) were filtered out using the “small segments rejection” procedure described by Poulsen et al. (2018). For the statistical analysis, we extracted the coverage parameter (the proportion of time covered by each microstate).

### ECG Analysis

Cardiac activity was recorded through an electrode placed below the left clavicle (Biosemi ActiveTwo System, Amsterdam, The Netherlands). The ECG preprocessing was performed using the EEGbeats (Thanapaisal, Trejo & Robbins, 2020) plugin for EEGLAB. To accurately identify the R-peaks and to minimize interference from noise and artifacts, an offline bandpass filter was implemented, set to a frequency range of 2 Hz to 27 Hz. The mean heart rate (HR) and the heart rate variability (HRV) measures, specifically the Root Mean Square of Successive Differences of inter-beat interval (RMSSD) and the High Frequency component (HF-HRV) were obtained.

### Statistical Analysis

All data analyses were conducted using the Jamovi software (v2.3.0, 2021). Initially, preliminary exploratory analyses were performed, including normality tests, identification of outliers, descriptive analysis, and distributions of the variables. To understand the relationships among the various measures used in this study (HEPs, HRV, microstates, and Body Perception Questionnaire scores), Spearman correlation analyses were conducted. The significance level was set at *p* <.05. Additionally, to complement these analyses and provide a more robust approach to false positives, Bayesian statistics were employed. Specifically, Bayes factors (BF_10_) were computed relative to the null hypothesis for the correlation analyses using Kendall’s tau.

## Results

### Microstate (MS) Analysis

Based on the cross-validation criterion, five distinct microstates (MS) were identified (See Fig. 1c). These five MS collectively explained 58.05% of the Global Explained Variance (GEV). Although the GEV observed is lower than values typically reported, it is similar to those found in previous studies using resting-state EEG (Hu et al. 2022; Seitzman et al. 2017). Based on their topographical features, aligning with descriptions in the existing literature, the MS were designated as maps A, B, C, D, and E (Michel and Koenig 2018; Tarailis et al. 2024).

### Relationships Between Self-Report and Psychophysiological Measures

Exploratory correlation analyses were conducted between the psychophysiological measures and the various subscales’ scores (i.e., Body Awareness, Subdiaphragmatic Reactivity, Supradiaphragmatic Reactivity, and Autonomic Reactivity) of the Body Perception Questionnaire (BPQ).

**Fig. 2:**
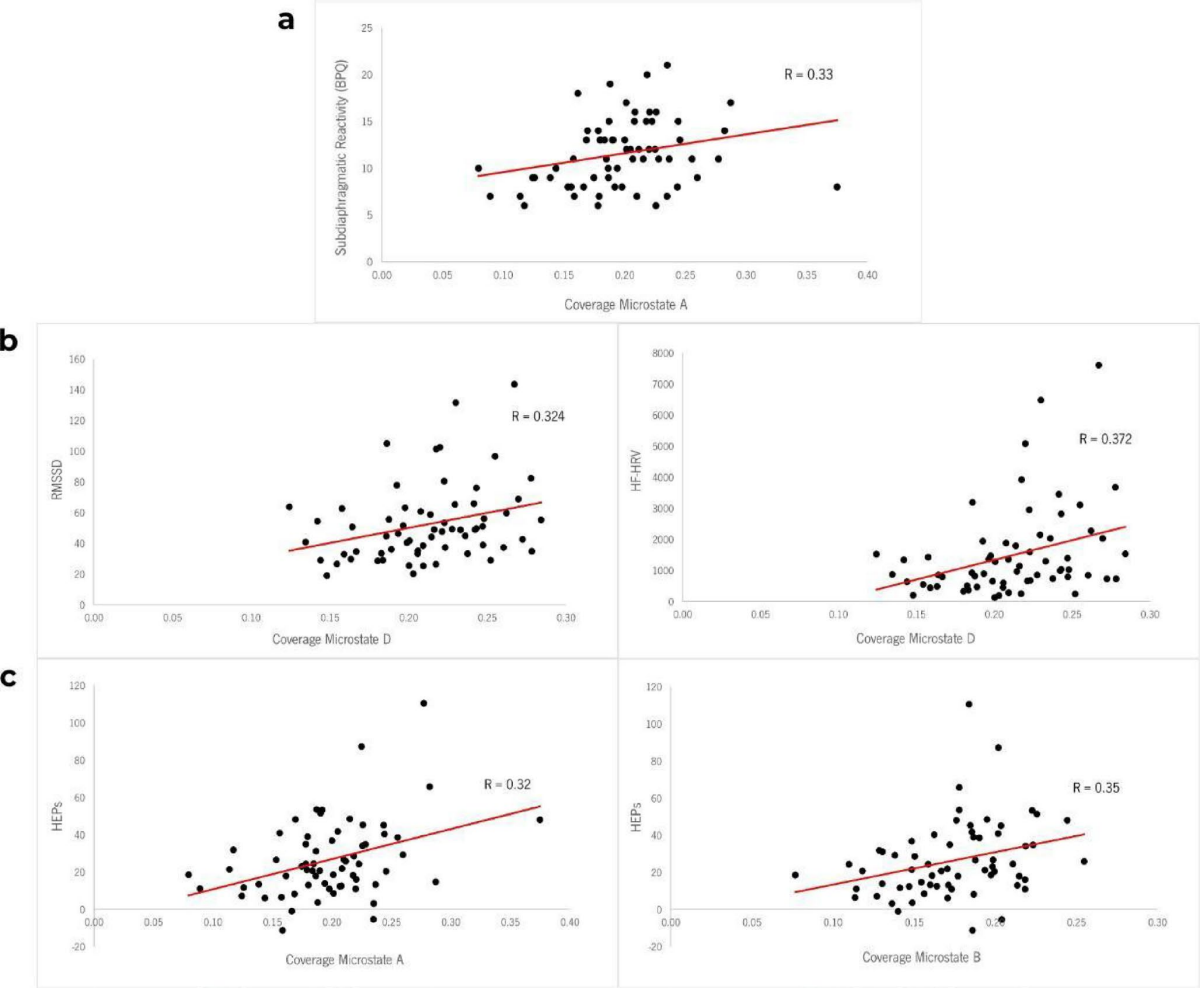
Significant correlations between microstates and other measured variables. **a**: Association between the coverage of Microstate A and the Subdiaphragmatic Reactivity subscale of Body Perception Questionnaire (BPQ). **b**: Association between the coverage of Microstate D and HRV measures (left– RMSSD, right– HF-HRV). **c**: Association between the coverage of Microstate A (left) and B (right) with Heartbeat Evoked Potentials (HEPs)

A positive correlation was found between the Subdiaphragmatic Reactivity subscale of BPQ and the coverage of MS A (Kendall’s τ = 0.226, *p* =.011; bayesian: 95%, CI [0.054; 0.377], BF_10_ = 4.98), indicating moderate evidence supporting an association between both indexes (See Fig. 2a). However, this correlation didn’t remain significant after false discovery rate (FDR) correction.

There is strong evidence of no correlation between the BPQ subscales and the maximum HEP amplitudes (Body Awareness: BF_10_ = 0.23; Subdiaphragmatic Reactivity: BF_10_ = 0.16; Supradiaphragmatic Reactivity: BF_10_ = 0.22; and Autonomic Reactivity: BF_10_ = 0.20), indicating that the self-reported interoceptive dimension was not related to the neurophysiological marker of interoception (HEPs). Similarly, no relationships were found between the BPQ scores and both HF-HRV (Body Awareness: BF_10_ = 0.16; Subdiaphragmatic Reactivity: BF_10_ = 0.19; Supradiaphragmatic Reactivity: BF_10_ = 0.27 and Autonomic Reactivity: BF_10_ = 0.35) and RMSSD (Body Awareness: BF_10_ = 0.17; Subdiaphragmatic Reactivity: BF_10_ = 0.17; Supradiaphragmatic Reactivity (BF_10_ = 0.41) and Autonomic Reactivity: BF_10_ = 0.45), indicating substantial evidence against the presence of an association.

### Relationships Between Cardiac Measures, HEPs and EEG Microstates

We investigated whether there were associations between the psychophysiological measures, including cardiac measures (RMSSD and HF-HRV), the heartbeat evoked-potentials (HEPs; mean and maximum peak amplitude), and the coverage of each EEG microstate (CovA, CovB, CovC, CovD and CovE).

Positive correlations were found between both HRV indices (RMSSD and HF-HRV) and the coverage of MS D (CovD) (See Fig. 2b). Specifically, RMSSD showed a positive correlation with CovD (Kendall’s τ = 0.224, *p* =.009; bayesian: 95% CI [0.052, 0.375], BF_10_ = 4.725), indicating that greater HRV as measured by RMSSD was associated with higher coverage of MS D. Similarly, HF-HRV also demonstrated a positive correlation with CovD (Kendall’s τ = 0.244, *p* =.004; bayesian: 95% CI [0.07, 0.395], BF_10_ = 8.804), providing moderate to strong evidence that an increase in parasympathetic activity, reflected in HF-HRV, corresponded to a greater coverage of MS D.

Regarding HEPs, the maximum HEP amplitude exhibited positive correlations with CovA (Kendall’s τ = 0.219, *p* =.010; bayesian: 95% CI [0.047, 0.371], BF_10_ = 4.077) and CovB (Kendall’s τ = 0.234, *p* =.006; bayesian: 95% CI [0.06, 0.385], BF_10_ = 6.407), providing moderate to strong evidence that higher maximum HEP amplitudes are associated with greater coverage of these MS (See Fig. 2c). Conversely, a negative correlation was found between the HEP amplitude and CovD (Kendall’s τ = − 0.175, *p* =.041; bayesian: 95% CI [−0.331, − 0.006], BF_10_ = 1.255), although with anecdotal evidence that higher HEP peak amplitudes were associated with reduced coverage of MS D. After applying FDR correction, none of the observed correlations remained statistically significant.

## Discussion

This study aimed to investigate whether self-reported interoceptive processes (BPQ scores) are related to several psychophysiological indices of interoception. Specifically, we analyzed autonomic markers of cardiac function (namely RMSSD and HF-HRV), indicators of cortical interoceptive processing of cardiac signals (HEPs), and distinct patterns of brain activity represented by EEG microstates. Our results suggest an association between the Subdiaphragmatic Reactivity subscale of BPQ and the coverage of microstate A. Contrary to our hypothesis that self-reported interoceptive dimensions from the BPQ would show associations with neurophysiological indices of interoception (such as HEPs and HRV), no correlations were found, suggesting that subjective interoceptive experience and these neurophysiological markers may reflect different facets of interoceptive processing. Our findings also suggest associations between HRV measures and the coverage of microstate D, as well as between HEP amplitudes and the coverage of microstates A and B.

Microstate A, assumed to be associated with auditory processing and arousal states, seems to be related to neural activity in temporal regions, as well as in the insular cortex (Britz et al. 2010; Custo et al. 2017; Tarailis et al. 2024). Our findings revealed an association between the coverage of microstate A and the Subdiaphragmatic Reactivity subscale of BPQ. This subscale predominantly measures reactivity to gastrointestinal sensations, such as bloating, digestion, or bowel activity (Kolacz et al. 2018). The insular cortex, particularly its posterior region, plays a crucial role in integrating interoceptive signals, including gastrointestinal sensations. Moreover, evidence has highlighted the insula’s role in functional gastrointestinal disorders, supported by brain imaging studies using experimental paradigms over recent decades (Van Oudenhove et al. 2010; Zeng et al. 2011). Therefore, this association might reflect the integration of subdiaphragmatic interoceptive signals, potentially mediated by the insular cortex, which is central to the processing of visceral sensations and the regulation of arousal states (Livneh and Andermann 2021; Nagai et al. 2021). Alternatively, this finding could indicate that individuals with higher subdiaphragmatic reactivity have an increased sensitivity to internal bodily states, which may enhance arousal levels and, consequently, the engagement of microstate A. This aligns with research indicating that interoceptive facets can influence arousal and emotional processing (MacCormack et al. 2024).

Contrary to our hypothesis that self-reported interoceptive sensibility (BPQ scores) would be associated with HRV and HEPs, no significant associations were observed. This absence of correlation may reflect the distinct - and sometimes non-overlapping - facets of interoception captured by self-report questionnaires and psychophysiological measures. Indeed, growing evidence supports the multidimensional nature of interoception, with several studies highlighting the relative independence of its various components (Mallorquí-Bagué et al. 2014).

Although the BPQ is based on individuals’ conscious awareness and subjective interpretation of bodily sensations - which are susceptible to imprecision and cognitive bias (Garfinkel et al. 2015) - even broader instruments like the Multidimensional Assessment of Interoceptive Awareness (MAIA), which assess emotional awareness, self-regulation, and body trust, have shown little to no association with HEPs (Baranauskas et al. 2017; Mai et al. 2018). Moreover, the relationship between interoceptive sensibility and HRV remains understudied, with limited empirical evidence available to support consistent links between these constructs. Factors such as sample characteristics (e.g., healthy vs. clinical populations), experimental context (e.g., resting-state vs. task-based paradigms), and statistical power likely influence the detectability of such associations. Notably, both HRV and HEPs reflect automatic and largely unconscious processing of interoceptive signals, relying on distinct autonomic and neural pathways that may not directly map onto self-reported experiences (Park and Blanke 2019).Reflecting the brain’s processing of cardiac signals, HEPs have been suggested as a key neurophysiological marker of interoception (Coll et al. 2021). Our results indicate that higher HEP amplitudes were associated with increased coverage of microstate B, which seems primarily involved in visual processing, self-awareness, self-visualization, and autobiographical memory. This microstate has also been linked to negative BOLD signals in posterior occipital regions (Britz et al. 2010; Tarailis et al. 2024). This result suggests a potential relationship between interoceptive processing and occipital network activity during the resting state. In this sense, previous studies showed that interoception has a significant impact on bodily self-awareness and self-identification (Candia-Rivera et al. 2024; Mehling et al. 2009). Additionally, within the framework of the Allostatic Interoceptive Network, cardiac activity is reported to play a crucial role in supporting the brain’s precision in perception and action (Skora et al. 2022). Specifically, signals originating from the heart have been shown to influence brain dynamics, behavior, and subjective experience, underscoring the importance of brain-heart interactions (Azzalini et al. 2019; Candia-Rivera 2022). For instance, studies have demonstrated that interoceptive stimuli can modulate (e.g., enhance or suppress) the processing of basic visual stimuli, suggesting that neural responses to heartbeats might be linked to the conscious experience of visual processing (Ronchi et al. 2017; Salomon et al. 2016). The framework of Allostatic Interoceptive Network is compatible with the predictive coding theory, which points out that the human brain forecasts and models external requirements using internal signs and demands as a basis (Migeot et al. 2022; Quigley et al. 2021). Sometimes, however, prediction errors occur when the anticipated model cue differs from the actual signal. This mismatch could help rectify future anticipations, thus being adaptive (Kleckner et al. 2017) or setting off dysfunctional responses (Quigley et al. 2021; Sennesh et al. 2022).

We also found a positive association between the coverage of microstate A and the HEP amplitude. This result aligns with evidence suggesting that the HEP is sensitive to different levels of arousal, with higher intrinsic levels being associated with a larger HEP (Luft and Bhattacharya 2015; Coll et al. 2021). Moreover, interoceptive signals such as heartbeats are known to influence the central nervous system’s arousal and alertness, potentially enhancing auditory readiness and sensory integration during resting states (Al et al. 2020). This dynamic interplay supports the adaptive functions of interoception, enabling seamless transitions between internal bodily awareness and environmental monitoring (Craig 2009; Garfinkel et al. 2016).

Positive associations were found between both RMSSD and HF-HRV and the coverage of MS D, indicating that greater HRV was associated with greater engagement of this MS. HRV is a well-established biomarker of autonomic nervous system flexibility and adaptability, with its parasympathetic components — particularly those captured by RMSSD and HF-HRV — reflecting vagal control over cardiac function. Higher HRV is associated with enhanced emotional regulation, cognitive control, and adaptability, reflecting the integrity of a central autonomic network that includes prefrontal regions and limbic structures (Balzarotti et al. 2017; Shaffer and Ginsberg 2017; Thayer et al. 2009). This network is critical for processing interoceptive signals by facilitating communication between brain regions responsible for monitoring and regulating bodily states (Smith et al. 2017; Critchley and Harrison 2013). Although the precise functions of each microstate are not yet fully understood, microstate D has been linked to attention-related processes and executive functioning (EF), with fronto-parietal networks identified as its neural underpinnings (Britz et al. 2010; Tarailis et al. 2024). Our results align with previous findings, suggesting a positive association between resting HRV indices and EF measures such as working memory, inhibition, and cognitive control (Forte et al. 2019; Williams et al. 2019). In fact, the vagus nerve may serve as a psychophysiological link between autonomic regulation and key brain areas, including the prefrontal cortex (PFC) and the anterior cingulate cortex (ACC), which play a crucial role in inhibitory control and self-regulation, both essential for EF. By influencing these brain areas, vagal control may enhance cognitive and emotional regulation, improving EF (Smith et al. 2017; Thayer et al. 2012).

Consistent with this proposal, evidence shows that the PFC seems to be strongly interconnected with key brain regions involved in interoceptive processes and emotional regulation, including the ACC and insula (Critchley et al. 2004; Seeley et al. 2007). These connections imply that the lateral PFC can access bodily sensation representations and play a top-down role in shaping interoceptive perception, highlighting the PFC’s role in coordinating responses to internal and external demands (Barrett and Simmons 2015; Etkin et al. 2011). Additionally, and in line with the predictive coding theory, interoceptive signals can be used to update goals or priors in the PFC, guiding the selection of actions that help maintain the body’s physiological states within an adaptive range (Dobrushina et al. 2021; Smith et al. 2017).

Taken together, these associations raise the possibility that resting-state brain dynamics, as captured by EEG microstates, may reflect ongoing interoceptive and autonomic processes. In particular, the link between HRV and microstate D might suggest that individuals with higher cardiac vagal activity - as estimated through HRV indices - engage more strongly in resting-state brain configurations related to attentional or regulatory functions. Similarly, the associations between HEPs and microstates A and B could indicate a relationship between heartbeat-related cortical processing and neural networks involved in arousal and sensory integration. Although these interpretations remain tentative, they are compatible with the predictive coding framework, which proposes that the brain continuously generates and updates predictions about internal bodily states in order to minimize interoceptive prediction errors. From this perspective, microstate dynamics could represent shifting prediction modes or updates within interoceptive networks, including the Allostatic Interoceptive Network (Santamaría-García et al. 2024).

A further interesting finding is that no associations were found between microstate E and the other variables analyzed. Although microstate E is often associated in the literature with salience and interoceptive processing (Pipini et al., 2017; Tarailis et al. 2021), particularly in relation to emotional and bodily awareness, it is possible that MS E’s involvement in interoception may depend on specific conditions (e.g., task-based paradigms) or individual differences. Moreover, previous research has suggested that the role of MS E may be more dynamic and context-dependent, possibly varying with specific emotional or sensory experiences rather than being uniformly linked to interoception (Tarailis et al. 2024).

It is important to note, however, that none of the reported correlations survived FDR correction for multiple comparisons. This limits the strength of the conclusions and suggests that the observed associations should be interpreted with caution.

## Limitations and Future Directions

The study’s cross-sectional design limits causal inferences and statistical power. Future research should include larger, longitudinal samples to confirm and expand upon these findings. The study of EEG microstates and their associated functions remains an emerging area of research, with many aspects still unclear (Damborská et al. 2019; Khanna et al. 2015). Furthermore, the interactions between interoceptive systems may be influenced by individual differences, such as transient emotional states and cognitive attention, which could shape how these measures are processed and related (Garfinkel et al. 2015). Thus, instead of capturing trait measures like those assessed by the BPQ, future studies could also assess participants’ emotional and cognitive states at a given moment in order to analyze how it influences the interplay between cortical and neurocardiac interoceptive processes.

Additionally, and as discussed by Vig and colleagues (2022), BPQ seems to translate a maladaptive interoceptive attention style associated with somatization, hypochondriasis, and anxiety, being weakly associated with other self-report measures of interoception (e.g., the Multidimensional Assessment of Interoceptive Awareness). This discrepancy between self-report measures should also be taken into account when considering these results. On the other hand, some of the BPQ subscales had highly skewed distributions, with many participants scoring very low, which likely reduced the variability necessary to detect meaningful relationships with the other measures. Furthermore, the interpretation of the BPQ items may be influenced by factors such as social desirability or difficulty in comprehending the nature of certain items, adding another layer of complexity to the assessment. These factors highlight the challenge of correlating self-reported interoception with physiological markers, as they may tap into distinct aspects of the interoceptive experience.

Given the hypothesis-driven nature of the analyses, multiple comparisons corrections were not applied. Instead, Bayesian methods were used to quantify the strength of evidence for each effect, providing a more nuanced and informative interpretation of the results (Efron 2005). Moreover, future studies should replicate this work using experimental tasks specifically designed to engage interoception, such as heartbeat detection or emotional regulation tasks, to better understand the dynamic role of interoception in cognitive and emotional processes. Extending this research to clinical populations, such as those with depression, anxiety disorders, or ADHD, would also be valuable, as these groups may show disruptions in the Allostatic Interoceptive Network and impairments in interoceptive processing (Kuhn et al. 2016; Kutscheidt et al. 2019; Santamaría-García et al. 2024).

Finally, while HRV was included as a measure of autonomic function, we did not control for respiration effects and other between-subject covariates (e.g., age, physical fitness), which may have influenced the results. Therefore, the interpretation of the HF-HRV component should be made with caution, as it is strongly influenced by respiratory parameters and may not exclusively reflect parasympathetic activity (Hayano and Yuda 2019; Heathers and Goodwin 2017).

## Conclusion

By integrating subjective, physiological, and neural data, this study aimed to advance our understanding of the mechanisms underlying the coordination of the brain and body within the framework of the Allostatic Interoceptive Network. Our results suggest an association between the Subdiaphragmatic Reactivity subscale of BPQ and microstate A, associated with auditory processing and arousal. We also found that HEPs were correlated with microstate B (associated with specific brain networks, including those related to visual processing and self-awareness) as well as microstate A (associated with auditory processing and arousal). Additionally, HRV measures were associated with microstate D, linked to attention-related processes and executive functioning. Self-reported interoception (BPQ) showed no significant associations with neurophysiological markers such as HEPs and HRV, indicating that subjective and objective measures of interoception may reflect different aspects of the process. This underscores the importance of considering interoception as a multidimensional construct.

## Data Availability

The data are available from the authors upon reasonable request.
